# Responsiveness of Nepali version of Oswestry Disability Index (ODI) on individuals with non-specific low back pain

**DOI:** 10.1186/s41687-021-00343-9

**Published:** 2021-08-09

**Authors:** Kandel Binaya, Thapa Kajal, Acharya S. Ranjeeta, Nepal Govinda

**Affiliations:** 1 Department of Physiotherapy, Kathmandu University School of Medical Sciences, Dhulikhel, Nepal; 2 Civil Service Hospital, Kathmandu, Nepal

**Keywords:** Disability evaluation, Minimal clinically important difference, Patient reported outcome measure, Reproducibility

## Abstract

**Background:**

Low back pain (LBP) is a common musculoskeletal problem, associated with disability and high societal costs. The Oswestry Disability Index (ODI) is among the most commonly used patient reported outcome measures to measure disability due to LBP. Evidence supporting the reliability and validity of the Nepali Version of Oswestry Disability Index (NODI) exists, but its responsiveness is yet to be assessed.

**Objective:**

We aimed to assess the responsiveness of NODI in participants with non-specific low back pain.

**Methods:**

The study included 102 (Male 41, Female 61) participants with non-specific low back pain, attending the physiotherapy outpatient department of a tertiary care hospital and nearby community. The NODI was administered to the patients at baseline and again 2 weeks later along with a 7-item Nepali Version of Global Rating of Change (GROC-NP). Responsiveness of NODI was assessed by plotting Receivers Operating Characteristics (ROC) curve.

**Results:**

The area under curve (AUC) of NODI was 0.88. The best cut-off point on the NODI for improvement on the GROC-NP or the minimal clinical important change (MIC) was 4.22 and ranged from 3.11 to 6.34. The sensitivity and specificity was 77.4% and 84.2% respectively.

**Conclusion:**

NODI is a responsive scale which can discriminate between participants whose level of disability due to LBP is stable or improving. The result for minimal clinically important change, sensitivity and specificity are consistent with other cross culturally adopted versions.

## Background

Low back pain (LBP) is the most common musculoskeletal pain leading to high treatment costs, absence from work and individual suffering [1]. It has an annual prevalence of 7.2% worldwide [2]. Globally, LBP is ranked highest in terms of disability and sixth in terms of overall individual suffering and economic burden [3] contributing to 10% of years lived with disability [4]. A global review of prevalence in a general adult population showed point prevalence of 12% to 33% and 1 year prevalence of 22% to 65% and lifetime prevalence ranged from 11% to 84% [5]. A study done in the US indicated that it was the second most common cause of disability in adults and a common reason for absenteeism, work lost and economic burden to the nation [6]. In the context of Nepal, the annual prevalence of back pain in Eastern Nepal has been reported as 71%, with prevalence of 67.9% in males and of 74.3% in females [7]. Patient with LBP report physical discomfort, low physical activity, functional limitations and decreased social participation. Such disability is measured by using patient reported outcome measures (PROMs) [8].

The Oswestry Disability Index (ODI) version 2.1a is the most commonly used outcome measure used to measure a patient’s permanent functional disability. The test is considered the ‘gold standard’ among low back functional outcome tools [9]. It is a self-administered questionnaire, comprising ten items assessing the extent of the patient’s back pain and their ability to carry out nine different activities of daily living. It has been translated and cross-culturally adapted into multiple languages [10] including the Nepali version (NODI) [11]. During the cross-cultural adaptation of the NODI, modification was made to the section “walking” where the units of measurement were changed from empirical (miles and yards) to metric (kilometers) to be consistent with the SI unit of length used in Nepal. The NODI has demonstrated excellent internal consistency (Cronbach’s alpha 0.72), test-retest reliability (intra-class correlation 0.87) and validity [11]. However, its responsiveness has not been assessed.

Responsiveness is a crucial characteristic of a measurement instrument and is defined as “the ability of an instrument to detect change over time in the construct to be measured” [12, 13]. The main goal of physical therapy is restoration of normal function and returning patients to their original functional status [13]. This requires measurement tools to assess functional status of a patient and to evaluate change in functional status over time [14, 15]. When a functional scale is used for evaluation of a treatment outcome, it should be able to detect small but important clinical changes i.e. should be “responsive” [16]. Responsiveness is thus important to evaluate treatment outcomes and for clinical decision making [17] by detecting clinical changes over time and interpreting the effect of clinical intervention [18, 19]. Nevertheless, responsiveness has is often neglected in the development of functional scales due to lack of standardization in methods, terminology and statistics [20]. Therefore the aim of this study was to investigate the responsiveness of the Nepali version of the ODI to determine its suitability for application in clinical and research settings.

## Methods

### Study design

The study was conducted at Dhulikhel Hospital, located in a sub-urban region of Nepal, 30 km from the capital city Kathmandu. Participants attending the physiotherapy Outpatient Department and nearby community were screened and included in this study. This research was conducted after the approval from the Institutional Review Committee, Kathmandu University School of Medical Sciences (IRC No 57/18). This study followed the Consensus-based Standards for the selection of health Measurement Instruments (COSMIN) guidelines [16]. Informed and/or written consent was obtained from all the participants prior to data collection.

We recruited participants with non-specific low back pain, aged 18 years or older with ability to understand and speak Nepali fluently from July 2018–May 2019. The participants were recruited by convenience sampling. Participants were excluded if they had history of a neoplasm, radiating pain from other sites, infections, systemic inflammatory disease, pregnancy, recent lumbar surgery (< 6 months) or specific low back conditions (eg. disc herniation, spondylolisthesis, recent fractures). Participation in this study was voluntary and those who didn’t provide consent were excluded. A sample exceeding 100 was set as an adequate number of participants [16].

The level of disability due to LBP was assessed at two different points of time, at an initial visit and at follow up assessment 2 weeks later. According to a Delphi study the time interval of 2 weeks was adequate to take two measurements [21]. Furthermore, the shorter duration (1–3 weeks) could avoid the possibility of recall bias associated with the use of the global rating of change scale [22]. A questionnaire package, consisting of a tool for screening inclusion and exclusion criteria and the NODI were administered on the initial visit for each participant**.** After 2 weeks, the NODI was re-administered along with the 7-item Nepali version of the Global Rating of Change (GROC-NP) to assess the participants’ perception of any change in their condition. The treatment administered was not taken into consideration in those 2 weeks, as this study’s intent was to evaluate the properties of the outcome measure and not the effectiveness of therapeutic intervention [23]. In order to avoid loss of follow up, phone call interviews were conducted for those who could not attend subsequent appointments.

### Outcome measures

Nepali Oswestry Disability Index (NODI) assesses the participant’s level of disability due to LBP. The questionnaire comprises of 10 items including nine everyday activities of daily living. The scoring is done by asking participants about their current functional status on a scale of 0–5. The total score is expressed as a percentage of maximal score, ranging from 0 to 100 with higher score indicating higher disability [9, 24].

Nepali Global Rating of Change (GROC-NP) measures the overall self-perceived change in a condition. It uses a likert scale with mid-point representing “no change”, a left anchor representing “very much worse” and a right anchor representing “very much better “or “recovered completely” [19]. Participant are asked to rate the difference between the initial and current level of disability due to LBP [25]. The level of the global perception of the condition can be collapsed to produce a dichotomous variable outcome: improved group (includes the levels described as completely better, much better and better) and not improved group (including the conditions little better, approximately the same thing, a little worse and very much worse) [19]. This method has been stated as standard for criterion based responsiveness calculation [16].

### Data analysis

Data was entered and analyzed using Statistical Package for Social Sciences version 16. Socio-demographic variables including age, sex, occupation, type of low back pain, treatment received, were reported using descriptive statistics using mean and standard deviation where applicable.

Minimal Clinical Important Change (MIC), was assessed using the anchor-based methods (criterion-based methods) [20]. GROC was used as an external criterion to assess responsiveness because of its high face validity [21]. This scale defined the change measured as “clinically important” as individuals graded their own health status. The change in NODI for each participant was calculated by subtracting the score at the follow up from the baseline score. A positive change score corresponded to improvement and negative score indicated deterioration of condition [26].

To differentiate between the improved group versus stable group, GROC-NP was used as an external anchor, assessed during the follow up visit [19]. Participants who chose “same as before”, a score of ‘4’ on GROC-NP were classified as the stable or unchanged group whereas participants who chose “slight improvement” ‘5’, “moderate improvement” ‘6’ or “a lot of improvement” ‘7’ were classified as responders. Participants who had worsening in their condition were classified as ‘deteriorated” (GROC-NP 3 or below) [26].

Responsiveness was evaluated in five steps as recommended by de Vet and colleagues [12],

- GROC-NP was used as the external anchor for the construct of interest (disability due to LBP assessed by using NODI).
- Individuals with LBP were chosen as the population of interest as they experienced varying levels of disability.
- We considered that the Area under Curve (AUC) of 0.70 or more as acceptable for the ability of NODI to differentiate between the groups that improved [13].
- The changes in scores of NODI over two time points were calculated with the independently collected GROC-NP scores, and
- Accuracy of the classification between changes in NODI scores and the responder/ stable categories were assessed using Receivers Operating Curve (ROC).

The ROC curve is a graph of “true positives” (sensitivity) versus “false positives” (1-specificity) for each of several cut-off points in change score [12]. It was interpreted as the probability of correctly discriminating between improved and non-improved groups. AUC theoretically ranges from 0.5 (no accuracy in discriminating improved from non-improved) to 1.0 (perfect accuracy) [27, 28]. The ROC curve provided an indication of the change score that represented the best cut-off threshold to discriminate between improved or not improved patients [8]. The optimal cut-off change score was identified when equally balanced sensitivity and specificity was found and considered as an expression of MIC. The Smallest Detectable Change (SDC) of the NODI was calculated using the following formula SDC = z x √2 x SEM, where z = 1.96 (z score for estimating a 95% confidence interval), √2 represents the two NODI measurements and SEM is the standard error of measurement [26].

## Results

One hundred and two participants were recruited for the evaluation of responsiveness of NODI. Fifty-six participants (54.9%) did not answer item 8 (sex life).

### Demographic characteristics

The demographic characteristics of participants in this study have been summarized in Table 1. Among the participants 41% were male and 61% were female with the mean age of 39 years. Most of the participants were engaged as housewives (35.3%), followed by sitting jobs and agricultural activities equally (18.6%). More than half of the participants (83%) had taken medication and were also taught physiotherapy exercises. Chronic LBP (76.5%) was reported at a much higher level than acute LBP (23.5%). The average follow-up duration among participants was 18 days.

**Table 1:** Demographic characteristics of the sample (*n* = 102)

Variables	N (%)	Mean (SD)
Age		39.7 (1.42)
Gender		
Male	41 (40.2)	
Female	61 (59.8)	
Occupation		
Agriculture	19 (18.6)	
Housewife	36 (35.3)	
Sitting job (business/office/student)	19 (18.6)	
Standing job	13 (12.7)	
Others	15 (14.6)	
Treatment Received		83 (81.4)
Types of low back pain		
Acute Low Back pain	24 (23.5)	
Chronic Low Back Pain	78 (76.5)	
Follow up duration in weeks		2.46 (0.63)

**Fig. 1:**
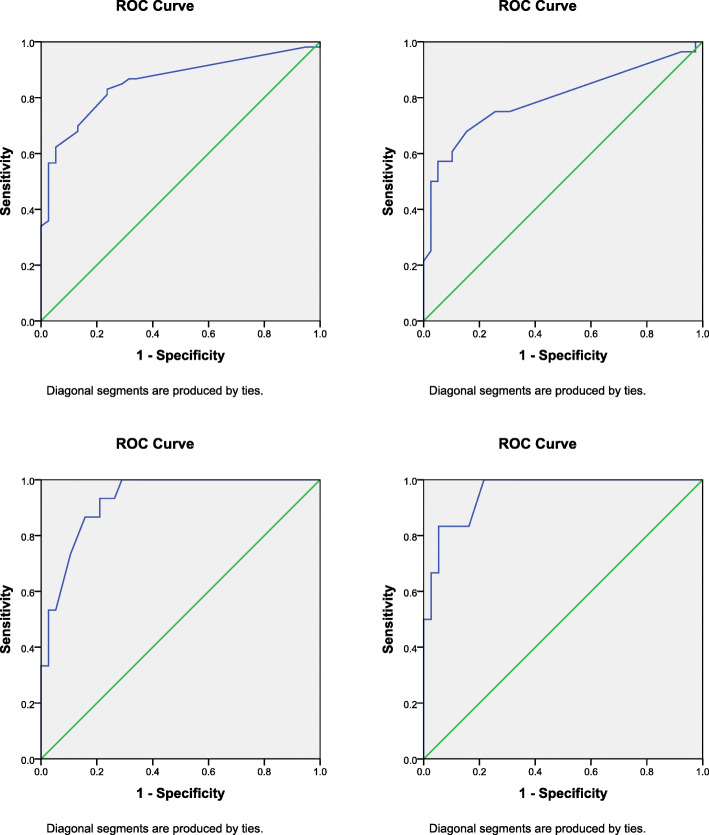
Receiver Operating Curve (ROC Curve) of stable (GROC-NP 4) Vs improved groups (GROC-NP 5, 6, 7)

The disability due to LBP, measured by using NODI in the initial and final assessment and the change in the level of disability is shown in Table 2. The findings obtained from GROC-NP, assessed in the follow up visit in all the 102 participants is illustrated in Table 3, where 91 participants (89%) showed either similar or improvement of their clinical condition. The ROC curve for NODI showing stable group (GROC-NP 4) versus the improved groups (GROC-NP 5, 6 and 7) and individually between the stable group and small (GROC-NP 5), medium (GROC-NP 6), large improvement (GROC-NP 7) is presented in Fig. 1. The AUC and MIC with sensitivity and specificity for each of these analyses is shown in Table 4.

**Table 2:** NODI Score in initial and final assessment

Nepali Oswestry Disability Index	Initial score Mean (SD)	Final score: Mean (SD)	Change Mean (SD)
Total (*N* = 102)	28.96 ± 14.94	23.19 ± 13.22	5.85 ± 9.32

**Table 3:** GROC-NP Interpretation

	N (%)	Mean ± SD
Same as before (GROC-4)	38 (37.3%)	1.40 ± 3.20
Slight improvement (GROC-5)	28 (27.5%)	7.41 ± 6.63
Moderate improvement (GROC-6)	15 (14.7%)	13.04 ± 11.90
A lot of improvement (GROC-7)	10 (9.8%)	16.76 ± 9.96
Deteriorated (GROC-3 and GROC-2)	11 (10.8%)	−2.11 ± 9.016

**Table 4:** Responsiveness of the NODI

	N	AUC (95%CI)	MIC	Sensitivity	Specificity	Mean change score ± SD
Primary Analysis (GROC 4 vs 5–7)	38/53	0.87	4.22	77.4%	84.2%	10.81 ± 9.60
Small improvement (GROC 4 vs 5)	38/28	0.79	3.11	71.4%	78.9%	7.50 ± 6.63
Medium improvement (GROC 4 vs 6)	38/15	0.93	5.22	86.7%	84.2%	13.034 ± 11.90
Large improvement (GROC 4 vs 7)	38/10	0.97	6.34	90%	94.7%	16.76 ± 9.96

*Abbreviations*: *NODI* Nepali version of Oswestry Disability Index, *GROC-NP* Global Rating of Change, *AUC* Area Under the Curve, *MIC* Minimum Important Change, *SD* Standard Deviation

The optimal cutoff score that was nearest to the upper-left corner of the ROC curve with the best combination of sensitivity and specificity (MIC) was 4.22. This value ranged from 3.11 to 6.34. The AUC was 0.87 and the value for sensitivity and specificity of NODI was 77.4% and 84.2% respectively. The value of SDC was 1.053. The MIC for “deteriorated” could not be calculated, as too few participants (*n* = 13).

## Discussion

This study aimed to assess the responsiveness of NODI on individuals with low back pain and showed the NODI to be a responsive scale with AUC of 0.87 and MIC value 4.22. The finding of this study is in line with other studies conducted to evaluate responsiveness of ODI. A literature search conducted by Michael Vianin on psychometric properties of ODI published in 2008 concluded that the values for the AUC ranged from 0.72 to 0.94, with MIC between 4 and 10.5 points and minimal change of 10 points was clinically significant [29]. Beurskens et al. reported a similar ROC value for ODI, AUC = 0.76 [25]. Taylor found that the ODI was more sensitive to patients who had improved and less sensitive for patients whose condition had remained unchanged [30]. The value of the AUC of the NODI is similar to that of other translated versions of ODI. The German and Chinese versions were found to be responsive to detect clinical change with an AUC of 0.90 and 0.77 [24, 31]. Similarly, the Brazilian–Portuguese version of the ODI had an AUC 0.73 and MIC value of 4.75 points [17]. The Italian version of ODI assessed on subjects with sub-acute or chronic low back pain had sensitivity of 76% and specificity of 63% [32].

The value of the MIC of the NODI was greater than the value of the SDC (1.053) and therefore, confirms the NODI can reliably detect change over time [8]. There is considerable confusion about responsiveness due to lack of clear standards for its measurement and interpretation [20]. Different methods are used to explore the responsiveness of an outcome measure, such as distribution-based methods and the criterion-based methods. Davidson and Keating concluded that, a distribution-based method provides no information about whether change is clinically meaningful, whereas a criterion-based method may be able to detect meaningful change in a clinical setting [8]. Hays et al. noted that: “only anchor based (criterion based) methods estimate whether group change is big enough to be regarded as clinically important. The so-called distribution-based indices are simply a way of expressing the observed change in a standardized metric [33]. Therefore, we used the Criterion based method as it gives an actual definition of MIC through an ROC analysis.

For evaluating responsiveness, we used GROC-NP measure as an external criterion, because change can be defined as “clinically important” if an individual grades their own health status [21]. Jaeschke et al.’s full definition of the MIC is: “the smallest difference in a score of a domain of interest that patients perceive to be beneficial and would mandate, in the absence of troublesome side effects and excessive costs, a change in the patients’ management” [26]. Taking this definition into consideration, the MIC greatly depends on the type of anchor and the anchor’s definition of important change. Some authors use “large improvement” as the standard to reflect minimally important improvement while others use “moderate improvement”, or even “slight improvement” to be the minimally important improvement as measured by the anchor [34].

In our study, 56 participants (54.9%) did not answer item 8 (sex life). Some of the participants were unmarried or lost their spouses or were too elderly and therefore considered this item not relevant to their life circumstances. Others were reluctant to answer as it is a culturally sensitive topic in Nepal and people hesitate to discuss their sex life. Similar findings have been reported on other Asian countries too [31, 35]. In such instance, if one section was missed or not applicable, the score was calculated by dividing the total score by 45 instead of 50 [9] and interpretation uses only nine domains.

### Strength and limitation

The strengths of this study included the variability among the participants representing both rural and urban areas and the large sample size of 102 participants.

Some of the limitations to be considered are:

1. Recall bias, the use of a retrospective global rating scale has been challenged. However, the short time follow-up in the current study (i.e.2 weeks) could reduce the possibility of obtaining a recall bias associated with the global rating scale [22].
2. No minimal clinically relevant difference for “deteriorated” could be calculated, as too few participants reported deterioration in their condition. A larger population based study may enable this measurement property to be determined [16].
3. This result can be generalized only in people with non-specific low back pain as responsiveness is a population-specific property [13].

## Conclusion

This is the first study to evaluate the responsiveness of the NODI in participants with LBP thus facilitating its use in both clinical and research setting. We used a criterion-based approach with the GROC-NP as the external criterion. Our results suggest that NODI is a responsive scale which can discriminate between participants whose level of disability due to LBP is stable or is improving. Hence it can be used as an evaluative tool to assess level of disability over time and to monitor the effects of treatment.

## Data Availability

The data sets used and/or analyzed during the current study are available from the corresponding author on reasonable request.

## Abbreviations

LBP: Low Back Pain
ODI: Oswestry Disability Index
NODI: Nepali Version of Oswestry Disability Index
GROC: Global Rating of Change
GROC-NP: Nepali Version of Global Rating of Change
ROC: Receivers Operating Characteristics
AUC: Area under curve
MIC: Minimal clinical important change
PROM: Patient reported outcome measures
IRC: Institutional Review Committee
SDC: Smallest Detectable Change
COSMIN: Consensus-based Standards for the selection of health Measurement Instruments
